# Globalization of Continuing Professional Development by Journal Clubs via Microblogging: A Systematic Review

**DOI:** 10.2196/jmir.4194

**Published:** 2015-04-23

**Authors:** Matthew John Roberts, Marlon Perera, Nathan Lawrentschuk, Diana Romanic, Nathan Papa, Damien Bolton

**Affiliations:** ^1^School of MedicineThe University of QueenslandBrisbaneAustralia; ^2^Centre for Clinical ResearchThe University of QueenslandBrisbaneAustralia; ^3^Mackay Base HospitalMackayAustralia; ^4^Urological Society of Australia and New ZealandEdgecliffAustralia; ^5^University of MelbourneDepartment of SurgeryAustin HospitalMelbourneAustralia; ^6^Olivia Newton-John Cancer Research InstituteAustin HealthMelbourneAustralia; ^7^Peter MacCallum Cancer CentreDivision of Cancer SurgeryMelbourneAustralia

**Keywords:** journal club, social media, continuing medical education, continuing professional development, systematic review

## Abstract

**Background:**

Journal clubs are an essential tool in promoting clinical evidence-based medical education to all medical and allied health professionals. Twitter represents a public, microblogging forum that can facilitate traditional journal club requirements, while also reaching a global audience, and participation for discussion with study authors and colleagues.

**Objective:**

The aim of the current study was to evaluate the current state of social media–facilitated journal clubs, specifically Twitter, as an example of continuing professional development.

**Methods:**

A systematic review of literature databases (Medline, Embase, CINAHL, Web of Science, ERIC via ProQuest) was performed according to Preferred Reporting Items for Systematic Reviews and Meta-Analyses (PRISMA) guidelines. A systematic search of Twitter, the followers of identified journal clubs, and Symplur was also performed. Demographic and monthly tweet data were extracted from Twitter and Symplur. All manuscripts related to Twitter-based journal clubs were included. Statistical analyses were performed in MS Excel and STATA.

**Results:**

From a total of 469 citations, 11 manuscripts were included and referred to five Twitter-based journal clubs (#ALiEMJC, #BlueJC, #ebnjc, #urojc, #meded). A Twitter-based journal club search yielded 34 potential hashtags/accounts, of which 24 were included in the final analysis. The median duration of activity was 11.75 (interquartile range [IQR] 19.9, SD 10.9) months, with 7 now inactive. The median number of followers and participants was 374 (IQR 574) and 157 (IQR 272), respectively. An overall increasing establishment of active Twitter-based journal clubs was observed, resulting in an exponential increase in total cumulative tweets (*R* ^2^=.98), and tweets per month (*R* ^2^=.72). Cumulative tweets for specific journal clubs increased linearly, with @ADC_JC, @EBNursingBMJ, @igsjc, @iurojc, and @NephJC, and showing greatest rate of change, as well as total impressions per month since establishment. An average of two tweets per month was estimated for the majority of participants, while the “Top 10” tweeters for @iurojc showed a significantly lower contribution to overall tweets for each month (*P*<.005). A linearly increasing impression:tweet ratio was observed for the top five journal clubs.

**Conclusions:**

Twitter-based journal clubs are free, time-efficient, and publicly accessible means to facilitate international discussions regarding clinically important evidence-based research.

## Introduction

Journal clubs are a well-established method to facilitate interactive peer review and critical thinking in clinical evidence-based medical education [1,2]. Furthermore, journal clubs provide a forum for academic debate and professional networking. Skills learned in critical analysis and literature appraisal skills are crucial in continuing professional development, in order to exercise best practices by any medical student or senior attending/consultant. Components of an effective journal club include regular and appropriate timing, high attendance rate (compulsory or incentive-based), nominated chairman, literature aligned with the journal club goals and reviewed prior to journal club session, and continuing professional development [3-6]. Unfortunately, many of these prerequisites act as limiting factors in a busy clinical practice.

An unprecedented expansion in the medical use of social media, such as Twitter, Facebook, LinkedIn, and YouTube, has followed the uptake seen with the general public. Twitter is a public, microblogging forum where users (each with a unique handle, eg @username) upload short messages comprising a maximum of 140 characters, with/or attached photos, documents, and links to other media such as videos, presentations, or journal articles. In most cases, these “tweets” are linked to a theme, often centralized using a “#” (hashtag, eg #twitter) for easy view and discovery by other users. Users are also able to “follow” the tweets of other users. Specific events (eg, medical conferences, public sporting events) often promote an official hashtag to allow users to follow all discussion relating to the event [7]. Dedicated explanation of social media apps in medicine and health care is available, with many falling under the broad banner of Free Open Access Medical education (FOAM) [8,9].

When combined with Twitter, journal clubs are able to function in a similar way to traditional journal clubs, with the advantage of a global audience and participation for discussion. Twitter-based journal clubs are able to be easily linked using a hashtag (eg, #...jc), allowing anyone to follow and contribute with a unique identifiable username. A central moderator is able to inform followers of the article to be discussed well ahead of time for perusal. Furthermore, authors of discussed articles are often invited as participants, enabling real-time interaction. Online journal clubs allow for international and increased participation, even when used with other less mainstream platforms [10,11]. Additional benefits of Web-based journal clubs include immediate feedback and discussion with authors and colleagues, as well as enhanced publication dissemination [1,12].

The aim of the current study was to evaluate the current state of journal clubs facilitated by social media, specifically Twitter, as an example of continuing professional development and through a systematic review process.

## Methods

### Overview

A systematic review was undertaken based on guidelines outlined by the Cochrane Collaboration and Preferred Reporting Items for Systematic Reviews and Meta-Analyses (PRISMA) statement [13].

### Published Literature Search

A systematic literature search was performed using literature databases (Medline via OVID, Embase, CINAHL, Web of Science, ERIC via ProQuest) using synonyms relating to “social media” and “journal club” in November 2014 (see Multimedia Appendix 1) [14,15]. Reference lists of the identified articles were also searched. Initially, synonyms relating to medical education were included but were later removed due to overexclusion of potential manuscripts. Included articles were published reports on the use of social media (specifically Twitter in order to investigate a specific platform) as a means of facilitating journal clubs. Following exclusion of duplicates, irrelevant articles, and abstracts of conference proceedings based on citations and full text, remaining articles meeting inclusion criteria were reviewed for methodology and summarized. Article selection was performed by 2 independent evaluators (MR, MP) and any discrepancies resolved.

### Twitter Hashtag Search

Following review of published reports, Twitter was by far the most popular and commonly used social media outlet for journal clubs. A systematic search of Twitter was performed to identify all relevant hashtags to be included in the current study, including using the search box and the terms “journal club” and “jc”, as well as reviewing the users who were following all identified journal clubs, initially using those identified in the literature search. Journal club selection was performed by 2 independent evaluators (MR, MP), and any discrepancies were resolved.

Inclusion criteria for hashtag analysis included journal clubs related to health care. Hashtags were excluded from analysis if they were not Twitter-based journal clubs, English-speaking, if the hashtag was not used completely for the purpose of a journal club, or if the hashtag represented institutional or private journal clubs. A final search was performed on the Symplur website, which tracks social media related to health care.

### Data Extraction

Basic Twitter-based data was extracted from the relevant journal club Twitter-based websites. If available, such information included speciality, location, journal club tweets, and Twitter followers. Journal club tweets refer to the number of posts generated by a single journal club account. Data extraction from Symplur was achieved by searching each relevant hashtag (#). Data extracted from Symplur included hashtag commencement date, hashtag inactivation date, total tweet count, tweet count per month, number of tweeters, and total impressions. “Hashtag activation date” was defined as first month with greater than 5 tweets with the relevant hashtag. “Hashtag inactivation date” was defined as the starting point of 3 consecutive months with <6 tweets per month. Tweet count refers to the total number of posts containing the relevant hashtag. Number of tweeters was defined as the number of unique individual Twitter accounts that generated a post containing the relevant hashtag. Impressions, or reach, refer to the number of Twitter users using a particular hashtag and the sum of their respective followers—thus a surrogate for the number of users exposed to a particular hashtag. Detailed statistics of the top five journal clubs (@NephJC, @igsjc, @EBNursingBMJ, @iurojc, and @ADC_JC), as determined by rate of increase in cumulative tweets, were retrieved for each month from Symplur.

### Statistical Analysis

Data from published manuscripts were insufficient for compilation, so a descriptive analysis was performed. Twitter hashtag data were collated and analyzed using a Microsoft Excel 2003 database. Figures were created using STATA v.12.0 SE.

## Results

### Literature Search

**Figure 1:**
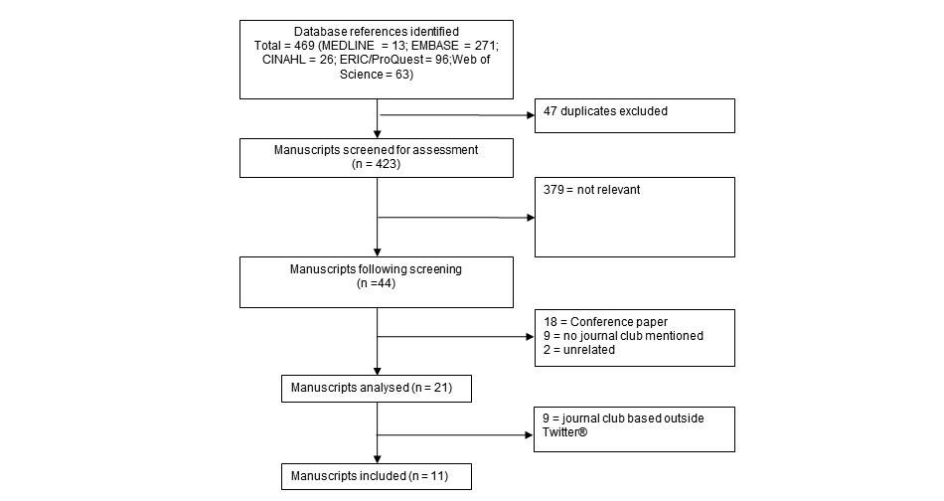
Flowchart of study selection as per the PRISMA statement.

The final search strategy resulted in retrieval of 469 citations, including 47 duplicates, 381 irrelevant citations, 18 conference abstracts, 9 citations unrelated to journal clubs, and 9 based on blogs or other resources (Figure 1). Eleven manuscripts were included and analyzed [1,11,16-24], which included reference to five Twitter-based journal clubs (#ALiEMJC, #BlueJC, #ebnjc, #urojc, #meded).

Three manuscripts referred to #ALiEMJC/@AnnalsofEM [16-18] (which reported the social media responses to journal club discussions), four to #BlueJC/@bluejchost [1,19-21] (comprising a narrative description [1] with accompanying letter to the editor and author reply [19,20], and inclusion in a narrative review [21]), one to #ebnjc/@EBNursingBMJ in a regional nursing publication raising awareness for #ebnjc [22], two to #urojc/@iurojc [11,23] (comprising a narrative description and analysis of the first 12 months [11] and letter to the editor regarding a recently discussed manuscript [23]), and one to #meded/@JournalGIM (as an editorial providing a narrative description [24]).

Of the manuscripts reviewed, three journal clubs were officially affiliated with peer-reviewed journals (#ALiEMJC/@AnnalsofEM to *Annals of Emergency Medicine*, #BlueJC/@bluejchost to *BJOG An International Journal of Obstetrics and Gynaecology*, #meded/@JournalGIM to *Journal of General Internal Medicine*), while the remainder have grown out of specialty interest groups and as yet appear to have no official alignment.

### Twitter Hashtag Search

Following the hashtag and Twitter-based journal club search, 34 potential hashtags/accounts were collated. Following review, 10 hashtags and associated journal clubs were excluded: three due to multiple uses for particular hashtag, two hashtags were related to private or institutional journal clubs, four were excluded due to complete inactivity or commenced within 1 month of assessment, and one was excluded due to non-English language. Of the 24 included hashtag accounts, the median months active was 11.75 (interquartile range [IQR] 19.9, SD 10.9; Table 1). Symplur analytic data were unavailable for three hashtag-related journal clubs.

**Table 1:** Demographic information for Twitter-based journal clubs, including specialty, frequency, commencement, inactive status, and associated peer-reviewed publications.

Hashtag, citation	Associated Twitter user	Speciality	Frequency	Commencement	Inactive date^a^
#1carejc^b^	@amcunningham	Primary care	NR	15/04/2013	5/6/2014
#ACCJournalclub^b^	@ACCinTouch	Cardiology	NR	29/3/2014	29/3/14
#ADC_JC	@ADC_JC	Pediatrics	NR	28/5/2014	Current
#ALiEMJC [16-18]	@AnnalsofEM	Emergency medicine	NR	15/11/2013	Current
#ambjc^b^	@ambjournalclub	Emergency medicine	Bimonthly	1/7/2011	11/11/13
#bluejc^b^ [1]	@bluejchost	Women’s health	NR	30/3/2013	Current
#cpjc^b^	@clinpsyJC	Psychology	NR	1/7/11	1/10/13
#ebnjc [22]	@EBNursingBMJ	Nursing	Bimonthly	5/1/2010	Current
#GeriMedJC	@gerimedJC	Geriatric medicine	Monthly	1/7/2014	Current
#HEJC	twubs/HEJC	Health economics	NR	1/9/2012	Current
#hpmJC	@hpmjc	Hospice, palliative medicine/care	NR	1/03/14	Current
#IGSJC	@igsjc	General surgery	Monthly	5/2/2014	Current
#jamapedsjc	@jamapeds	NR	21/10/2014	8/8/14	Current
#JC_StE^b^	@JC_StE	Emergency medicine	Bimonthly	1/10/2012	1/10/13
#MedEd^c^ [24]	@JournalGIM	General medicine	NR	NR	
#microtwjc	@microtwjc	Microbiology	NR	11/9/2013	Current
#NephJC	@Nephjc	Nephrology	NR	20/4/2014	Current
#PGHANJC	@BSPGHAN	Pediatric gastroenterology	NR	2/6/2014	
#PHTwitJC	@PHTwitJC	Public health	Monthly	1/7/2011	1/8/13
#psychjc	@psychiatryjc	Psychiatry	Monthly	28/9/2014	Current
#rsjc	@respandsleepjc	Respiratory	Monthly	26/6/2014	Current
#swjcchat^b^	@swjcchat	Social work	Bimonthly	7/7/2013	1/12/13
#twitjc^b^	@twitjournalclub	General medicine	Bimonthly	1/5/2011	1/12/13
#urojc [11]	@iurojc	Urology	Monthly	1/10/2012	Current

^a^If applicable, defined as 3 consecutive months of fewer than 5 tweets per month.

^b^Inactive journal clubs.

^c^#MedEd is not a unique hashtag for this journal club—it is also used for discussion among other medical educators.

### Online Journal Club Activity

Of the 24 included journal club–related hashtags, the median number of followers was 374 (IQR 574) with a median number of active participants of 157 (IQR 272). Monthly activity was calculated with a median “tweets per month” of 203 (IQR 317) and median “impressions per month” of 165,538 (IQR 504,654). Following inception, and as of October 30, 2014, seven of the included journal clubs had become inactive (#1carejc, #ambjc, #cpjc, #JC_StE, #PHTwitJC, #swjcchat, #twitjc).

**Figure 2:**
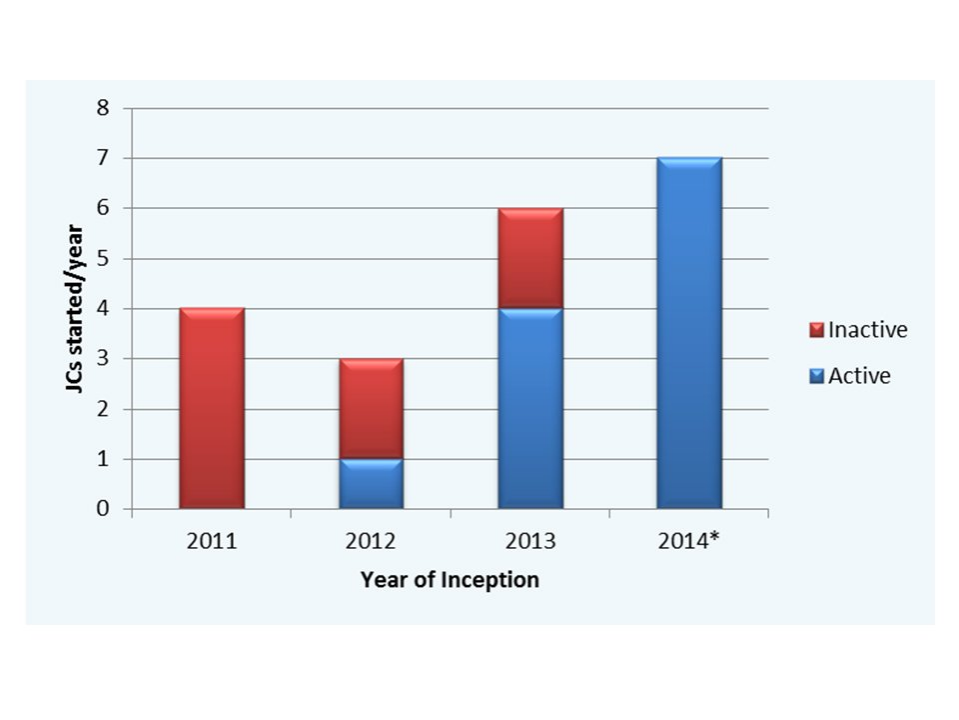
Establishment of journal clubs per year, comparing all journal clubs (blue) with currently active journal clubs (red). 2014 included journal clubs started prior to October 2014.

Overall, after exclusion of inactive journal clubs, an increasing establishment of Twitter-based journal clubs (Figure 2) was observed. Furthermore, an exponential increase in cumulative tweets was observed (*R* ^2^=.98) as well as tweets per month (*R* ^2^=.72).

For specific journal clubs, a continual increase in cumulative tweets in the early (<24 months) stages was observed. Specifically, the linearly modeled (all *R* ^2^>.95) increase in tweets was estimated as being greatest for @NephJC (722 tweets/month), @igsjc (613 tweets/month), @EBNursingBMJ (417 tweets/month), @iurojc (345 tweets/month), and @ADC_JC (255 tweets/month). These trends were also observed in the impressions/month rankings (Table 2). The two journal clubs that have previously been the longest running but now inactive, @twitjournalclub and @PHTwitJC, showed an inverse exponential cumulative tweet relationship following very high initial activity. Individually, no definite trends in tweets per month were observed, owing to large fluctuations in monthly activity.

**Table 2:** Twitter-based journal club performance, incorporating standard metrics such as tweets and followers, as well as overall tweets, participants and impressions, with calculated monthly mean tweets and impressions relating to each journal club as defined by Symplur*.*

Hashtag	Associated Twitter user	Tweets	Followers	Total tweets	Mean tweet/mo^a^	Participants	Impressions/mo^a^
#1carejc^b^	@amcunningham	NA	NA	564	41	171	191,161
#ACCJournalclub^b^	@ACCinTouch	NA	NA	NA	NA	NA	NA
#ADC_JC	@ADC_JC	1452	459	2785	546	142	378,763
#ALiEMJC	@AnnalsofEM	NA	NA	924	80	234	136,145
#ambjc^b^	@ambjournalclub	57	87	93	3	42	1380
#bluejc	@bluejchost	516	202	3705	194	290	126,455
#cpjc^b^	@clinpsy*JC*	67	201	216	5.4	61	18,039
#ebnjc	@EBNursingBMJ	2117	1399	3901	395	456	719,241
#GeriMedJC	@gerimedJC	130	158	318	80	52	36,044
#HEJC	twubs/HEJC	NR	NR	986	38	103	18,176
#hpmjc	@hpmjc	588	129	1694	212	176	223,686
#IGSJC	@igsjc	624	750	5199	586	430	843,358
#jamapedsjc	@jamapeds	NR	NR	387	140	76	535,852
#JC_StE^b^	@JC_StE	615	374	1008	84	73	58,346
#MedEd^c^	@JournalGIM	NA	NA	NA	NA	NA	NA
#microtwjc	@microtwjc	525	155	NA	NA	NA	NA
#NephJC	@Nephjc	1436	584	5295	832	478	1,184,105
#PGHANJC	@BSPGHAN	NA	NA	NA	NA	NA	NA
#PHTwit*JC*	@PHTwit*JC*	1817	1057	4245	170	320	139,916
#psychjc	@psychiatryjc	92	72	240	218	44	288,109
#rsjc	@respandsleepjc	929	176	1669	401	115	86,730
#swjcchat^b^	@swjcchat	758	481	1199	138	138	6144
#twitjc^b^	@twitjournalclub	1498	3446	12,628	407	1,954	883,543
#urojc	@iurojc	1832	2401	9040	362	1,567	622,139

^a^During active period only.

^b^Inactive journal clubs.

^c^#MedEd is not a unique hashtag for this journal club—it is also used for discussion among other medical educators.

### Determinants of Journal Club Performance

**Figure 3:**
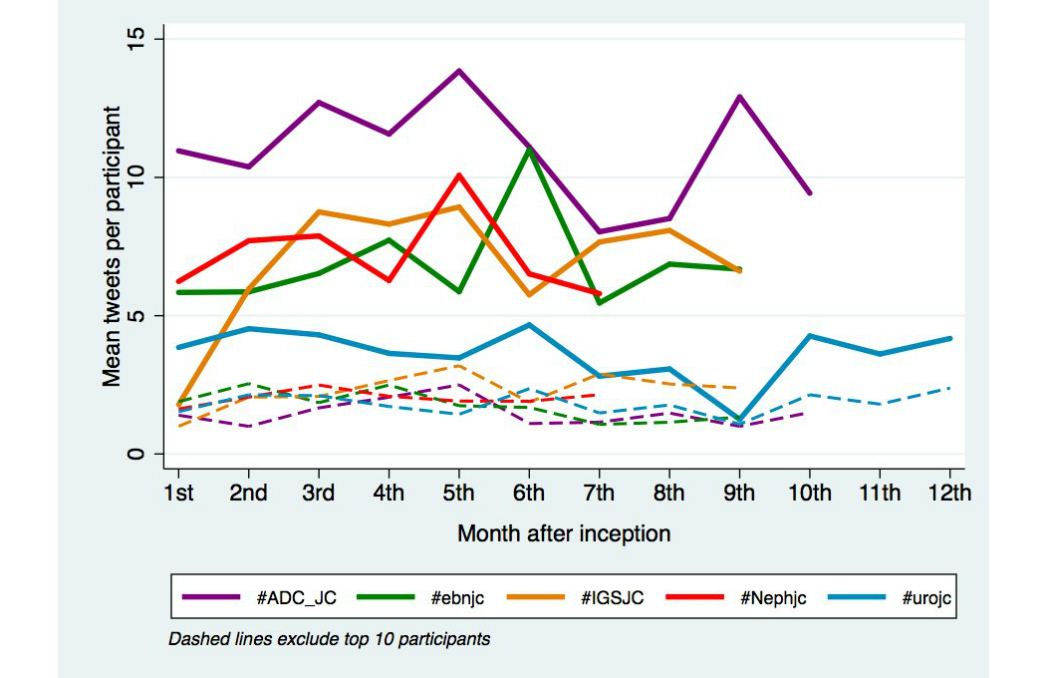
Average tweets per participant for the top 5 journal clubs (#ebnjc, #iurojc, #Nephjc, #ADC_JC, #igsjc). The overall calculated average for each journal club is represented by solid lines, while the average for participants outside of the Top 10 is represented by dashed lines.

Tweets and impressions for each of the top five journal clubs as determined by tweets/impressions per month (ie, @ADC_JC, @EBNursingBMJ, @igsjc, @iurojc and @NephJC) were analyzed. Subgroups were created based on the “Top 10” participants for each month (as determined by Symplur) versus the remaining participants. An average of two tweets per month, was estimated for participants outside of the “Top 10” tweet contributors for each month (Figure 3). The contributions of the “Top 10” to overall tweets were significantly lower for #urojc (*P*<.005). Of those appearing in the “Top 10” for each month during journal club discussions, the majority were classified in the “Top 10” for the first or second time.

**Figure 4:**
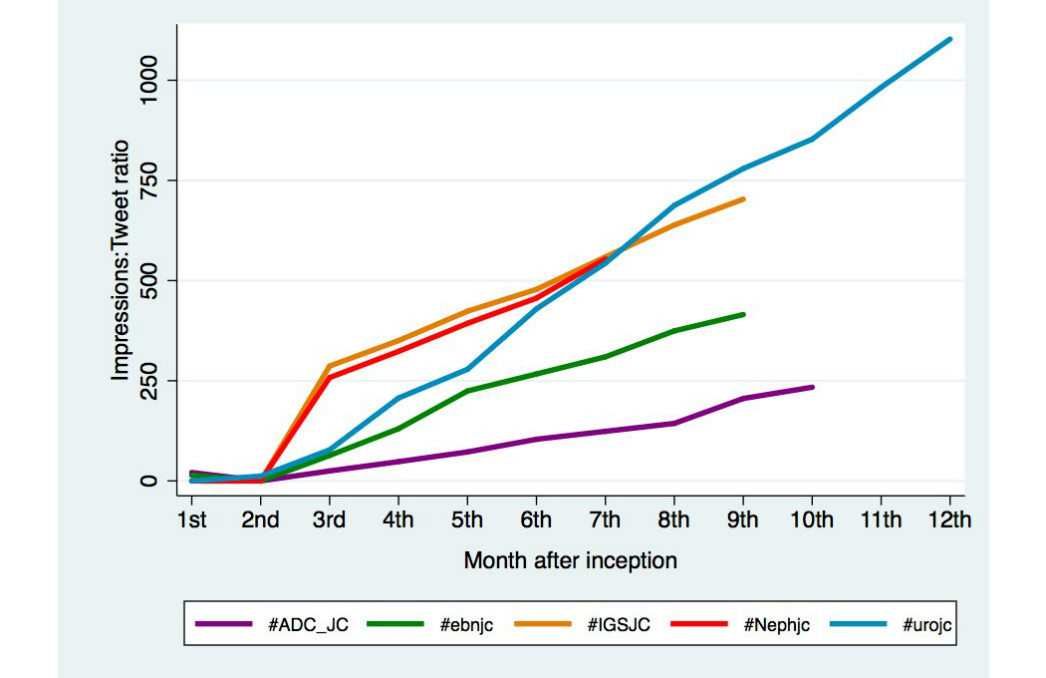
Journal club user account impressions:tweets ratio as a measure of reach, represented as absolute ratio change per month.

In an attempt to measure “reach” for each journal club, while considering large tweet traffic from these accounts in moderating and advertising, an impression:tweet ratio was calculated for each journal club user account. This impression:tweet ratio was shown to be linearly increasing for all journal clubs (*R* ^2^>.87 for all; Figure 4).

## Discussion

### Principal Findings

There were an estimated 200 million users per month actively tweeting an average of 500 million times per day in 2013, depicting Twitter as a contemporary, ever-changing social media environment. While recent reports in media outlets suggest that the rate of Twitter use overall may be declining, the findings of this systematic review of published literature and Twitter suggest that Twitter-based journal clubs are an expanding method of continuing professional development and a platform for global interaction. Published reports describe positive initial uptake and support from their respective medical communities [11,16-18], while Twitter analytics confirm an increasing trend in journal club establishment, cumulative tweets, and tweets per month.

Overall, we have observed an increasing participation in Twitter by the medical and allied health community, evidenced by an increasing year-to-year establishment of new journal clubs (Figure 2) and exponential increase in cumulative tweets and impressions. While the increase in tweets and impressions may be a function of journal clubs being established, it is more likely reflective of an ever-increasing global audience, reflected by the increasing impressions:tweets ratio for all journal clubs analyzed (Figure 4), and involvement of a wider, global audience viewing and participating in these discussions. Although the benefits of international involvement cannot be overemphasized, the benefits to local and regional formats may also be beneficial in communicating best practice or regional guidelines for countries and health jurisdictions where geographic separation is a significant obstacle. Given the current information explosion in medical research, Twitter also represents a potentially credible alternative to traditional “Commentary” pieces in peer-reviewed journals, allowing input from multiple key opinion leaders not previously available.

Similar increases in Twitter participation have been observed for medical conferences [7] and established peer-reviewed journals [25]. This increasing involvement by the global health audience appears to benefit traditional peer-review establishments. For journals, this increase in audience exposure appears to be beneficial for performance indices, with Twitter profiles associated with a higher mean impact factor for journals [25]. Furthermore, tweets have been reported to be predictive of future citations, with highly tweeted articles 11 times more likely to be highly cited than less tweeted articles in one journal [26].

While this relationship between social media and traditional academic media continues to grow, the use of Twitter for continuing professional development is an attractive venture. However, the freedom of voluntary participation complicates the establishment of an accurate and efficient record of participation for appropriate ethical acknowledgement for continuing professional development requirements by credentialing authorities. To date, no objective evaluation assessing the knowledge uptake and retention resulting from microblogging journal club is available [11]. Some strategies could include a posttest, similar to that provided by peer-reviewed journals, or a survey distributed by Twitter and completed online. These strategies may also be valuable in assessing participation in Free Open Access Medical Education (FOAM), which has also been facilitated by social media (eg, @UrologyQuiz found on Twitter).

FOAM encapsulates a collection of resources and tools for learning in medicine that are transforming medical education [9]. FOAM has continued to expand using social media as different collaborative teaching resources in accordance with popularity of online digital media, including blogs, podcasts, tweets, Google hangouts, online videos, and Facebook groups [27]. Twitter-based journal clubs are another addition to the FOAM sphere, available on Twitter (using hashtag #FOAMed). These resources allow individuals to upload medical content with discussion, collaboration, and dissemination of knowledge among users occurring on the individual site. The FOAM movement is a contemporary approach to improving and adding new collaborative resources to Web-based medical/health education, continuing professional development and research services [28]. Critics of FOAM have suggested that because such resources can be easily published online without quality control mechanisms, unreviewed FOAM resources may be erroneous or biased [29]. Peer-review processes for FOAM publications have been recently implemented in the form of high subject expertise from clinicians as either a pre-publication review and linked blog post for further comments, or post-publication review and facility for the author to amend or respond to the expert comments [29]. The Social Media Index (SM-i) is an emerging comparative index tool for FOAM resources, which combines quantitative online data to provide an overall rank to be calculated for any FOAM site [30], similar to other established peer-reviewed instruments comparing scholars (h-index), journals (Impact Factor), and websites (HONcode, DISCERN).

When individual journal club performance was considered, we observed a clear increase in overall tweets and impressions for established journal clubs, with some increasing at a rate as high as 722 tweets/month. However, month-to-month tweeting was observed to be highly variable. This variability could relate to diversity in interest among followers regarding the topics discussed or reduced participation of influential or high-volume tweeters for various reasons. There may also be limitations in access to articles discussed, particularly for individuals without institutional or individual journal access for those articles that are not open access. Where possible, some journal clubs, such as #urojc, provide open access to the discussed articles in order to remove this limitation [11].

In considering the determinants of journal club performance, subgroup analysis suggested that a large proportion of tweets each month were from those in the “Top 10”, with the remaining participants expected to contribute two tweets per journal club discussion (Figure 3). However, the majority of users appearing in the “Top 10” for each month during journal club discussions were doing so for the first or second time. This observation suggests that the broader audience participation and occasional generation of high traffic by eager participants outweighs the influence of regularly active tweeters or key Twitter-opinion leaders, who promoted the journal clubs to gain an initial following. However, in this open environment, there is also potential to overload the discussion or self-promote, creating unnecessary “noise”. This focus on gaining followers and impressions as well as “mentions”, which are similar to citations of a peer-reviewed manuscript may be strategic in improving a user’s Klout score, an overall social media popularity rating [25]. Furthermore, the significantly lower contribution from the “Top 10” for #urojc compared to other journal clubs may be a consequence of longer establishment of #urojc, thus gaining a broader contribution to the discussion. The observed increasing impressions:tweets ratio suggested that the reach of the journal clubs analyzed continues to grow with time, resulting an in ever-expanding audience for these high-level academic discussions.

### Limitations

This study is an analysis of social media at a point in time, when social media is known to be ever evolving. Much of the Twitter-based analytics relied on third-party services, such as Symplur, which was intended to focus on social media related to health care. Further, there are inherent limitations with the use of Twitter-based outcome measures, such as impressions, as a surrogate for reach. We were unable to measure the pattern of growth in followers or participants through data acquisition restrictions. There is also no current way to measure the passive value of journal clubs, specifically relating to users who are following the journal club discussion, and thus acquiring educational value, but not actively participating. We acknowledge that the number of included manuscripts is small, reflecting the current state of published literature relating to Twitter-based journal clubs. This review will serve as a check point and reference for the development of enhanced Twitter-based journal clubs by other medical craft groups internationally or loco-regionally.

### Conclusions

This systematic review provides an illustration of early trends in the development of journal clubs using Twitter as a communication medium. Twitter-based journal clubs provide access to free, time-efficient, and high-level discussions on clinically important issues and equal participation opportunity for users. Twitter provides an unprecedented method of networking and formation of friendships with colleagues, which can be harnessed to educate, initiate research collaborations, and even canvass opinions with difficult cases in the time between conferences. Hence, the role of social media in continuing professional development will continue to evolve with increasing engagement by journals, conferences, and FOAM sources. Furthermore, in the midst of busy clinical duties, microblogging using Twitter provides a unique pathway to access and engage in discussions with peers and professional leaders.

## Multimedia Appendix 1

Terms used for search of literature databases.

## Abbreviations

FOAMed: Free Online Access Medical Education
PRISMA: Preferred Reporting Items for Systematic Reviews and Meta-Analyses
