# Supplementary material for: Globalization of Continuing Professional Development by Journal Clubs via Microblogging: A Systematic Review
Source: J Med Internet Res. 2015 Apr 23;17(4):e103. doi: 10.2196/jmir.4194 (PMC4424319; doi:10.2196/jmir.4194)
Supplement: Supplementary file 1 [file jmir_v17i4e103_app1.pdf]

Supplementary Table 1: Terms used for search of literature databases

MEDLINE (via OVID)		EMBASE (via OVID) -		CINAHL (via EBSCO)		ERIC via ProQuest		Web of Science (All databases)	
social media.mp. or exp Social Media/	1928	'social media'/exp	2910	TX social media* OR TX social network* OR TX facebook* OR TX twitter* OR TX blog* OR TX webcast* OR TX podcast*	16259	(social media* OR social network* OR facebook* OR twitter* OR blog* OR webcast* OR podcast*) AND (medical education* OR resident education* OR medical student education* OR continuing medical education* OR evidence* based medicine*) AND (journal* club*)	3	**TOPIC:** (social media* OR social network* OR facebook* OR twitter* OR blog* OR webcast* OR podcast*) Timespan=All years Search language=Auto	340558
social network*.mp.	8231	social AND media*	59478						
exp Social Networking/	945	social AND network*	32776						
facebook*.mp.	678	Facebook*	1382						
twitter*.mp.	423	twitter*	822						
exp Blogging/ or blog*.mp.	1005	blog*	1805						
exp Webcasts as Topic/ or webcast*.mp.	678	webcast*	434	TX medical education* OR TX resident education* OR TX medical student education* OR TX continuing medical education* OR TX evidence* based medicine*	34785	**(social media* OR social network* OR facebook* OR twitter* OR blog* OR webcast* OR podcast*) AND (journal* club*)**	**96**	**TOPIC:** (medical education* OR resident education* OR medical student education* OR continuing medical education* OR evidence* based medicine*) Timespan=All years Search language=Auto	682146
podcast.mp.	235	podcast*	529						
1 or 2 or 3 or 4 or 5 or 6 or 7 or 8	11332	#1 OR #2 OR #3 OR #4 OR #5 OR #6 OR #7 OR #8	90480						
medical education.mp. or exp Education, Medical/	140966	medical AND education*	527236						
exp "Internship and Residency"/ or resident education.mp.	37164	resident AND education*	18827	S1 AND S2 AND S3	4				
exp Faculty, Medical/ or exp Students, Medical/ or exp Education, Medical, Undergraduate/ or medical student education.mp.	44098	medical AND student AND education*	53996	**S1 AND S3**	**26**			**TOPIC:** (journal* club*) Timespan=All years Search language=Auto	2538
exp Education, Medical, Continuing/ or exp Evidence-Based Medicine/	78568	continuing AND medical AND education*	29517					#3 AND #2 AND #1 Timespan=All	13

						years<br><br>Search language=Auto	
10 or 11 or 12 or 13	210957	evidence* AND based AND medicine*	177049			#3 AND #1 Timespan=All years Search language=Auto	**63**
9 and 14	444	#9 OR #10 OR #11 OR #12 OR #13	691546				
journal* club*.mp.	994	#8 AND #14	9411				
15 and 16	5	journal AND club	18943				
**9 and 16**	**13**	#15 AND #16	53				
		**#8 AND #16**	**271**				
